# Disease reservoirs: from conceptual frameworks to applicable criteria

**DOI:** 10.1038/emi.2017.65

**Published:** 2017-09-06

**Authors:** Luisa K Hallmaier-Wacker, Vincent J Munster, Sascha Knauf

**Affiliations:** 1 Work Group Neglected Tropical Diseases, Pathology Unit, German Primate Center, Leibniz Institute for Primate Research, Kellnerweg 4, Göttingen 37077, Germany; 2 Primate Genetics Laboratory, German Primate Center, Leibniz Institute for Primate Research, Kellnerweg 4, Göttingen 37077, Germany; 3 Virus Ecology Unit, Laboratory of Virology, Division of Intramural Research, National Institute of Allergy and Infectious Diseases, National Institutes of Health, Rocky Mountain Laboratories, Hamilton, MT 59840, USA

**Keywords:** disease eradication, infection, infectious diseases, interdisciplinary, one health, multidisciplinary

## Abstract

Central to the One Health approach and any disease eradication program is the question of whether a pathogen has a non-human reservoir. Despite well-established conceptual frameworks that define a reservoir of infection, empirical characterization of reservoirs often remains controversial, challenging and sometimes misleading. What is essentially missing are applicable requirements that standardize the use of the term ‘reservoir of infection’ across multiple disciplines. We propose an empirical framework, considering maintenance and feasible transmission of a pathogen, to standardize the acceptance of a disease reservoir across multiple disciplines. We demonstrate the intended use of these requirements by applying them to different diseases that are known to infect both humans and animals.

## A RESERVOIR NEEDS TO MAINTAIN THE PATHOGEN AND HAVE A FEASIBLE TRANSMISSION ROUTE

**Figure 1:**
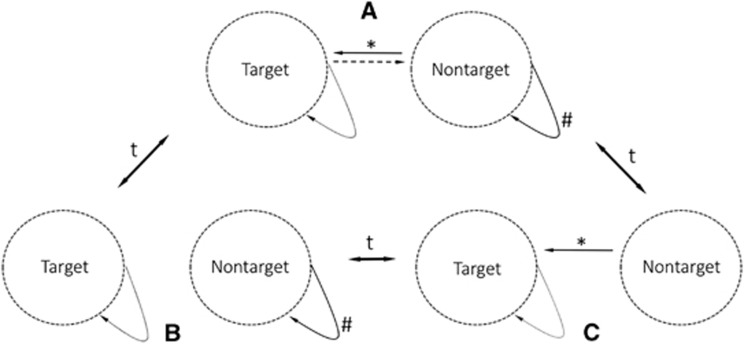
Three scenarios describing the dynamics of a simple reservoir system. (**A**) Pathogen maintenance in the non-target population and feasible transmission route towards the target population. Only this constellation fulfills the requirements of a functional reservoir system. (**B**) Pathogen maintenance in the non-target but no feasible transmission route towards the target population. This is a likely situation whether contact rates between the non-target and target populations are below the threshold. (**C**) No pathogen maintenance in the non-target, but a feasible transmission route exists. An example of the effect of a vaccination strategy in the non-target population. The dynamic of the system is indicated by arrows associated with a ‘t’ (time factor). ^#^Maintenance, *feasible transmission, solid arrows=obligatory, broken line=optional.

**Figure 2:**
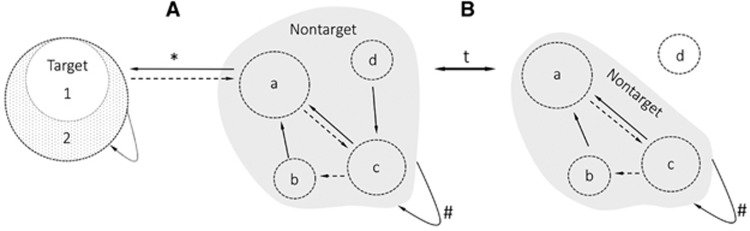
The simplicity and functional orientation of the presented framework allows for straightforward application but does not negate more complex populations. The same principles apply to multi-species systems and metapopulations. The defined target group may be adjusted based on interest and may therefore include metapopulations (targets 1 and 2). The non-target group increases in complexity due to the inclusion of multiple populations (a–d). (**A**) Similarly, to a simple reservoir system, all susceptible populations that connect to the target either (a) directly or (b–d) indirectly are part of the non-target population. (**B**) Temporal shifts in the ecological landscape of the non-target population may lead to the (d) exclusion of populations either due to lack of connectivity or susceptibility. The dynamic of the system is indicated by arrows associated with a ‘t’ (time factor). ^#^Maintenance, *feasible transmission, solid arrows=obligatory, broken line=optional.

The high prevalence of infectious agents of zoonotic and anthropozoonotic origin pose a major health threat to both human and animal populations. A conceptual framework for understanding a reservoir of infection has been established through various studies that have emphasized different aspects of zoonotic diseases.^1, 2, 3, 4^ However, empirical characterization of reservoirs often remains controversial and challenging. The most applicable and accepted way to investigate and define a reservoir emphasizes the annotation of a target group (Figure 1), which is an explicitly defined population of interest in a dynamic and heterogeneous landscape (for example, humans at the livestock–wildlife–human interface).^4, 5^ According to Haydon *et al.*,^4^ the target group is a matter of definition and may therefore be disconnected from the ecological reality. The target group provides a directionality to the study of a reservoir system. All other susceptible populations (non-target populations), which directly or indirectly connect epidemiologically to the target (Figures 1 and 2), can be part of the potential reservoir.^4^ For a non-target population to be considered an accepted functional reservoir, maintenance of a single pathogen in the population needs to be shown in combination with a feasible transmission route between the target and non-target populations.^4^

Although the conceptual framework of a disease reservoir is already well-defined, applicable requirements for an evidence-based rejection or acceptance of a reservoir are currently missing. In particular, interdisciplinary standards on genetic and functional similarities of reservoir and human isolates of pathogens are nonexistent. Considering the increase in interdisciplinary research, we see the need to critically discuss and standardize the use of the term ‘reservoir of infection’ across different research fields to oppose the tendency of published scientific data to exaggerate positive results and hype certain areas of science.^6, 7^ Although we do not claim absolute standardization of empirical requirements to accept a reservoir across disciplines, we present a framework to serve as a basis for a pending discussion in the growing One Health community. The simplicity and functional orientation of the presented framework allows for straightforward application but does not negate more complex populations, as the same principles can be applied to multi-species systems and metapopulations (Figure 2).

According to the accepted definition of a reservoir proposed by Haydon *et al.*,^4^ we discuss the requirements in two parts: the pathogen’s maintenance in a potential population or community followed by a discussion on proof of a feasible transmission route. Although the two components are addressed separately, only together they demonstrate the existence of a functional reservoir.

## PROOF OF PATHOGEN MAINTENANCE IN A POTENTIAL RESERVOIR

Increases in technological advancements (for example, next-generation sequencing) and vast quantities of available data have not led to concrete applicable criteria when examining the capacity of a pathogen to be maintained in a population. Recognizing both the ethical limitations in regards to animal testing^8^ and the advances in the molecular detection of pathogens, we propose the following criteria to demonstrate the maintenance of a pathogen in a population: (i) a high-genetic similarity of the pathogen found in the reservoir system, (ii) a high degree of functional similarity (infectivity and viability), and (iii) a longitudinal approach that considers the factor of time (Table 1). Owing to the functional orientation of the requirements and for simplicity, all entities involved in the biological lifecycles of a parasite (for example, primary and intermediate hosts) should be considered a single functional unit. Appropriate sequence and functional analysis of a pathogen isolated multiple times from a potential reservoir should be required to prove that a pathogen is maintained in a population. The ability to quickly and cheaply sequence whole genomes has allowed for better genetic resolution.^49, 50^ Sequence data can be used to examine similarity in the pathogen between a potential reservoir and a target. However, mutation rates vary significantly between pathogens^51, 52^ and the threshold for sequence and functional similarity must be individually defined and accepted by the scientific community. A single-nucleotide difference can potentially result in a loss of infectivity, for example, when important invasion mechanisms are affected (receptor affinity). In bacteria, investigations can be further complicated by plasmids that can be exchanged and mutated over time.^53^ A high amount of phylogenetic relatedness of pathogens isolated from the non-target and target populations does not provide sufficient evidence for the involvement of a pathogen and its ability to infect both groups. Importantly, DNA-based analyses only provide information on the functional potential of a pathogen and must not reflect the gene-expression within a host.^54^ For example, the bacterium *Treponema paraluiscuniculi* (which causes syphilis in rabbits), is over 99% identical on the basis of the whole genome to the human pathogen *T. pallidum* (which causes human treponematosis), but does not infect humans.^55^ As phylogenetic information fails to reflect the downstream effects of mutations, proof that a pathogen can proliferate in the potential reservoir is required.^56^ Information on the transcriptome and proteome of bacteria or the phenotype of viruses are necessary to see the effect of mutations on pathogen viability.^57^ There are different ways to test for the functional ability of a pathogen in different species. Owing to the ethical concerns, cell and tissue assays have been increasingly used in therapeutic research instead of animal models.^8^ Although these assays are limited in their conclusiveness, they can provide important insight into the molecular mechanisms involved. For example, the failure to infect primary tissue culture from rhesus macaques with human immunodeficiency virus 1 (HIV-1) demonstrates that non-human primates were unlikely to act as a maintenance population (Table 1).^58^ In some instances, for example, with uncultivable bacteria such as *Treponema pallidum*, it may be necessary to use animal models to examine the functionality of a pathogen within a potential reservoir species. Knowledge of the biology of the pathogen is essential to properly define a sequence and functional similarity threshold for a particular reservoir system.

When examining pathogen maintenance, a longitudinal approach is required to consider the dynamics of a potential reservoir system, including the influence of genetic variation in any given population. Defining a population that was infected at a single time point as a maintenance population for a pathogen is based on assumptions and is therefore speculative. Sero-prevalence surveys are an attractive way to detect the presence of pathogens in a population, as it indicates that an immunocompetent subject was in contact with the pathogen.^1^ However, only longitudinal studies with adequate sampling regimes (multiple sampling) to test for antibodies against a pathogen can provide information on the timing or frequency of infection, both of which are important for reservoir studies.^59^ Furthermore, cross-reactivity and erroneous assays can lead to false-positive results. For more diffuse reservoir systems, including multi-species compositions where the diversity of host susceptibility (at the individual, species or population level) protects against widespread infection (dilution effect),^60^ a longer time frame must be applied. This guarantees a more accurate understanding of the maintenance within a population (for example, Ebola^36^).

## PROOF OF FEASIBLE TRANSMISSION ROUTE

Maintenance of a pathogen in a population alone does not provide sufficient proof that a functional reservoir exists. A connection between the target and the non-target populations must be established;^4^ otherwise the non-target population remains a maintenance population with the potential to be a reservoir. Therefore, the determination of a feasible and somewhat permanent transmission route between the non-target and target populations is key to identifying a reservoir system (Figure 1). For multi-species reservoir systems, the transmission route between the target and non-target populations may be indirect (Figure 2, connection between b and target), possibly incorporating different hierarchical levels of a non-target community.^4, 61^ The type of transmission route dictates the form of evidence needed to prove that a feasible transmission route exists between the reservoir and target. For simplicity, we define vectors as part of the transmission route, although under certain circumstances (for example, permanency or substantial amplification in the vector), they may act as part of the non-target community.^61^ Four basic requirements need to be met to make a compelling argument for the existence of a feasible transmission route: (i) spatial (direct or indirect) and temporal connectivity between the reservoir system and the target population, (ii) pathogen involvement in this feasible transmission route, (iii) proof of viability of the pathogen during the proposed transmission route and (iv) a longitudinal approach that requires the isolation of a pathogen multiple times in a given transmission route (Table 1).

To prove the feasibility of a transmission route, direct or indirect spatial connectivity as well as temporal overlap between the non-target and target populations must be present. Connectivity measurements depend on the type of transmission route; for example, direct contact transmission requires overlapping territory. Computational tools can help determine the necessary overlap in a population by modeling the transmission across an affected population.^62^ In addition to spatial and temporal overlap, the involvement of the pathogen in the particular transmission route needs to be shown, which again requires long-term field projects. In the case of Lyme disease caused by *Borrelia burgdorferi*, nucleic acids from the bacterium were detected in ticks using PCR.^44^ However, the detection of DNA does not directly prove that transmission occurs. To gain further confidence that the transmission is feasible, it is therefore essential to show that the infectious organism remains viable during the proposed transmission route.^45^ This means that in addition to PCR detection, the viable pathogen needs to be isolated during a transmission event, where the measure of viability depends on the type of pathogen. In airborne transmission, for example, environmental factors such as size of droplets, UV light and humidity can greatly influence the transmissibility of a virus (as reviewed in Tang^63^). If the amount of viable and therefore infectious organisms is below the infectious dose, the particular transmission route is unfeasible. Without a feasible transmission route between target and non-target populations, no functional reservoir exists. Furthermore, to include all parts of a reservoir population, long-term investigations must focus on the transmission between the non-target and target groups as well as feasible transmission within the non-target community.^61^ Unconnected maintenance host populations may become a future reservoir through temporal shifts of the ecosystem.

## CHALLENGES OF IMPLEMENTATION

Biological systems are dynamic and can change over time (Figure 1). Single transmission events do not confirm a reservoir of infection (for example, HIV,^20^ Table 1). It is therefore important to show continuity and persistence in both maintenance and transmission, which can only be achieved through multiple and adequately timed (field) investigations. Well-designed intervention studies can be used as quasi-experiments to study a reservoir of infection but should not be used as a stand-alone test for the existence of a reservoir.^1^ Despite sufficient planning, the cause and effect of intervention studies are often difficult to determine^1, 64^ and the removal of a pathogen from a particular ecosystem may cause unanticipated effects. A negative outcome does not necessary indicate the lack of a reservoir or transmission route.^64, 65^ Instead, it can show that the intervention may have been incomplete or that the complexity of a reservoir is not entirely understood.

Pathogens must be studied in the context of natural ecosystems. The complexity of reservoir systems increases as multiple non-target populations interact as an ecological entity, which is influenced by factors such as competition, co-existence or predation.^66^ Furthermore, the artificial environment in a laboratory, which is often used to study the susceptibility of a species, differs substantially from a natural setting.^67^ The use of laboratory animals or cell- and tissue-based assays can be advantageous when studying pathogenicity, but it cannot solely contribute to the understanding of the epidemiology of a pathogen, which is largely impacted by variables such as genetic diversity, co-infection, cross-protective immunity and spatial connectivity. As a consequence, any epidemiological model requires additional information on the geographic range and the ecological landscape.^68^ This includes population densities and functional profiles of species that are involved in the reservoir system.^60, 69^ The importance of sample size in field studies and animal experiments cannot be stressed enough as it greatly affects the efficacy of analysis, especially in reservoirs with low-frequency crossover events.

Neither laboratory experiments, nor intervention studies, nor epidemiological models alone can provide a full understanding of a natural reservoir of infection. Only the combination of methods that are based on established and validated species-specific assays and technically sound field investigations can provide confidence that the pathogen is maintained in a non-target population and that a feasible transmission route exists. This, however, requires the political will and financial support to conduct long-term One Health studies to explore diseases in their natural context.

## CONCLUSION

The term ‘disease reservoir’ should be used carefully and only if there is convincing evidence demonstrating the maintenance and a feasible transmission route of a particular pathogen (Figure 1). We propose overarching requirements that must be fulfilled to provide ample proof that a reservoir exists (Table 1). Classical reservoir systems (for example, Lyme disease caused by *Borrelia burgdorferi*) fulfill all of the requirements proposed in this study, whereas some well-known diseases, such as Ebola, need further research until a reservoir system can be accepted (Table 1). For the pathogens without an accepted reservoir, the framework introduced in this study also indicates the outstanding questions that future research should focus on to investigate the presence of a reservoir system. A broader expert-based multidisciplinary discussion is needed to develop standards for the diversity of pathogens.

## Figures and Tables

**Table 1:** Applicable requirements that need to be fulfilled for the acceptance of a disease reservoir and their exemplary use in selected diseases that are known to infect humans and animals

				**Maintenance in NT**		**Feasible transmission route**			**Time factor**	
**Pathogen**	**Target**	**Non-target**	**Main transmission route**	**High-genetic similarity**	**Functional similarity**	**Spatial and temporal connectivity**	**Pathogen involvement**	**Maintaining pathogen viability**	**Example of longitudinal study**	**Refs.**
Influenza A virus (H1N1)	Human	Swine	Aerosol	X	X	X	X	X	9	10, 11, 12
MERS-Coronavirus	Human	Camel	Direct contact	X	X	X	X	(X)	13	14, 15, 16
*Brucella melitensis* (localized brucellosis)	Human	Sheep	Food-borne	X	X	X	X	X	17	18, 19
Immunodeficiency virus	Human	NHP	Direct contact	(X)	NP	X	NP	NP	N/A	20
*Treponema pallidum pertenue* (yaws)	Human	NHP	Direct contact/vector	X	(X)	X	(X)	NP	N/A	21, 22, 23
*Mycobacterium bovis* (bovine tuberculosis)	Human	Cattle	Food-borne/aerosol	X	X	X	X	X	24	25, 26
Rabies virus	Human	Fox	Bite	X	X	X	X	X	27	28, 29, 30
*Echinococcus multilocularis* (alveolar echinococcosis)	Human	Fox	Oral/fecal	X	X	X	X	X	31	32, 33
Hantavirus	Human	Rodent	Aerosol	X	X	X	X	X	34	35
Ebola virus	Human	Bats	Contact/aerosol	X	NP	X	(X)	NP	N/A	36, 37, 38
Zika virus	Human	NHP	Vector	(X)	NP	X	X	NP	39	40, 41, 42
*Borrelia burgdorferi* (borreliosis)	Human	Wildlife	Vector	X	X	X	X	X	43	44, 45, 46
Yellow fever virus	Human	NHP	Vector	X	X	X	X	X	47	48

Abbreviations: not available, N/A; non-human primate, NHP; non-target, NT; not provided/no current evidence, NP; evidence, X; partial evidence, (X).

Classical reservoir systems fulfill all requirements proposed in this study.

## References

[bib1] Viana M, Mancy R, Biek R et al. Assembling evidence for identifying reservoirs of infection. Trends Ecol Evol 2014; 29: 270–279. 24726345 10.1016/j.tree.2014.03.002 PMC4007595

[bib2] Drexler JF, Corman VM, Müller MA et al. Bats host major mammalian paramyxoviruses. Nat Commun 2012; 3: 796. 22531181 10.1038/ncomms1796 PMC3343228

[bib3] Ashford R. When is a reservoir not a reservoir? Emerg Infect Dis 2003; 9: 1495–1496. 14725261 10.3201/eid0911.030088 PMC3035539

[bib4] Haydon DT, Cleaveland S, Taylor LH et al. Identifying reservoirs of infection: a conceptual and practical challenge. Emerg Infect Dis 2002; 8: 1468–1473. 12498665 10.3201/eid0812.010317 PMC2738515

[bib5] Dunning JB, Stewart DJ, Danielson BJ et al. Spatially explicit population models: current forms and future uses. Ecol Appl 1995; 5: 3–11.

[bib6] Caulfield T, Sipp D, Murry CE et al. Confronting stem cell hype. Science 2016; 352: 776–777. 27174977 10.1126/science.aaf4620

[bib7] Vinkers CH, Tijdink JK, Otte WM. Use of positive and negative words in scientific PubMed abstracts between 1974 and 2014: retrospective analysis. BMJ 2015; 351: h6467–h6777. 26668206 10.1136/bmj.h6467 PMC4677695

[bib8] Ferdowsian HR, Beck N. Ethical and scientific considerations regarding animal testing and research. PLoS One 2011; 6: e24059. 21915280 10.1371/journal.pone.0024059 PMC3168484

[bib9] Simon G, Larsen LE, Dürrwald R et al. European surveillance network for influenza in pigs: surveillance programs, diagnostic tools and Swine influenza virus subtypes identified in 14 European countries from 2010 to 2013. PLoS One 2014; 9: e115815. 25542013 10.1371/journal.pone.0115815 PMC4277368

[bib10] Brookes SM, Núñez A, Choudhury B et al. Replication, pathogenesis and transmission of pandemic (H1N1) 2009 virus in non-immune pigs. PLoS One 2010; 5: e9068. 20140096 10.1371/journal.pone.0009068 PMC2816721

[bib11] Steel J, Staeheli P, Mubareka S et al. Transmission of pandemic H1N1 influenza virus and impact of prior exposure to seasonal strains or interferon treatment. J Virol 2010; 84: 21–26. 19828604 10.1128/JVI.01732-09 PMC2798408

[bib12] Maines TR, Jayaraman A, Belser JA et al. Transmission and pathogenesis of swine-origin 2009A (H1N1) influenza viruses in ferrets and mice. Science 2009; 325: 484–487. 19574347 10.1126/science.1177238 PMC2953552

[bib13] Müller MA, Meyer B, Corman VM et al. Presence of Middle East respiratory syndrome coronavirus antibodies in Saudi Arabia: a nationwide, cross-sectional, serological study. Lancet Infect Dis 2015; 15: 559–564. 25863564 10.1016/S1473-3099(15)70090-3 PMC7185864

[bib14] Mohd HA, Al-Tawfiq JA, Memish ZA. Middle East respiratory syndrome coronavirus (MERS-CoV) origin and animal reservoir. Virol J 2016; 13: 87. 27255185 10.1186/s12985-016-0544-0 PMC4891877

[bib15] Van Doremalen N, Bushmaker T, Munster V. Stability of Middle East respiratory syndrome coronavirus (MERS-CoV) under different environmental conditions. Euro Surveill 2013; 18: 20590. 24084338 10.2807/1560-7917.es2013.18.38.20590

[bib16] Haagmans BL, van den Brand JM, Raj VS et al. An orthopoxvirus-based vaccine reduces virus excretion after MERS-CoV infection in dromedary camels. Science 2015; 351: 77–81. 26678878 10.1126/science.aad1283

[bib17] Dean AS, Crump L, Greter H et al. Global burden of human brucellosis: a systematic review of disease frequency. PLoS Negl Trop Dis 2012; 6: e1865. 23145195 10.1371/journal.pntd.0001865 PMC3493380

[bib18] de Figueiredo P, Ficht TA, Rice-Ficht A et al. Pathogenesis and immunobiology of brucellosis: review of Brucella–Host Interactions. Am J Pathol 2015; 185: 1505–1517. 25892682 10.1016/j.ajpath.2015.03.003 PMC4450313

[bib19] Paixão TA, Roux CM, Den Hartigh AB et al. Establishment of systemic *Brucella melitensis* infection through the digestive tract requires urease, the type IV secretion system, and lipopolysaccharide O antigen. Infect Immun 2009; 77: 4197–4208. 19651862 10.1128/IAI.00417-09 PMC2747930

[bib20] Gao F, Bailes E, Robertson DL et al. Origin of HIV-1 in the chimpanzee Pan troglodytes troglodytes. Nature 1999; 397: 436–441. 9989410 10.1038/17130

[bib21] Knauf S, Dahlmann F, Batamuzi EK et al. Validation of serological tests for the detection of antibodies against *Treponema pallidum* in nonhuman primates. PLoS Negl Trop Dis 2015; 9: e0003637. 25803295 10.1371/journal.pntd.0003637 PMC4372418

[bib22] Knauf S, Raphael J, Mitjà O et al. Isolation of *Treponema* DNA from necrophagous flies in a natural ecosystem. EBioMedicine 2016; 11: 85–90. 27488881 10.1016/j.ebiom.2016.07.033 PMC5049926

[bib23] Harper KN, Knauf S. Treponema pallidum infection in primates: clinical manifestations, epidemiology, and evolution of a stealthy pathogen. In: Brinkworth JF, Pechenkina K (eds). Primates, Pathogens, and Evolution. New York, NY: Springer, 2013: 189–219.

[bib24] Walker TM, Lalor MK, Broda A et al. Assessment of *Mycobacterium tuberculosis* transmission in Oxfordshire, UK, 2007–12, with whole pathogen genome sequences: an observational study. Lancet Respir Med 2014; 2: 285–292. 24717625 10.1016/S2213-2600(14)70027-X PMC4571080

[bib25] Fitzgerald S, Kaneene J. Wildlife reservoirs of bovine tuberculosis worldwide: hosts, pathology, surveillance, and control. Vet Pathol 2013; 50: 488–499. 23169912 10.1177/0300985812467472

[bib26] Michel AL, Müller B, van Helden PD. *Mycobacterium bovis* at the animal-human interface: a problem, or not? Vet Microbiol 2010; 140: 371–381. 19773134 10.1016/j.vetmic.2009.08.029

[bib27] Sidwa TJ, Wilson PJ, Moore GM et al. Evaluation of oral rabies vaccination programs for control of rabies epizootics in coyotes and gray foxes: 1995–2003. J Am Vet Med Assoc 2005; 227: 785–792. 16178403 10.2460/javma.2005.227.785

[bib28] Rupprecht CE, Hanlon CA, Hemachudha T. Rabies re-examined. Lancet Infect Dis 2002; 2: 327–343. 12144896 10.1016/s1473-3099(02)00287-6

[bib29] Hemachudha T, Laothamatas J, Rupprecht CE. Human rabies: a disease of complex neuropathogenetic mechanisms and diagnostic challenges. Lancet Neurol 2002; 1: 101–109. 12849514 10.1016/s1474-4422(02)00041-8

[bib30] Chautan M, Pontier D, Artois M. Role of rabies in recent demographic changes in red fox (*Vulpes vulpes* populations in Europe. Mammalia 2000; 64: 391–410.

[bib31] Schweiger A, Ammann RW, Candinas D et al. Human alveolar echinococcosis after fox population increase, Switzerland. Emerg Infect Dis 2007; 13: 878–882. 17553227 10.3201/eid1306.061074 PMC2792858

[bib32] Veit P, Bilger B, Schad V et al. Influence of environmental factors on the infectivity of *Echinococcus multilocularis* eggs. Parasitology 1995; 110: 79–86. 7845716 10.1017/s0031182000081075

[bib33] Craig P. *Echinococcus multilocularis*. Curr Opin Infect Dis 2003; 16: 437–444. 14501996 10.1097/00001432-200310000-00010

[bib34] Kuenzi AJ, Douglass RJ, Bond CW et al. Long-term dynamics of Sin Nombre viral RNA and antibody in deer mice in Montana. J Wildl Dis 2005; 41: 473–481. 16244056 10.7589/0090-3558-41.3.473

[bib35] Bi Z, Formenty PB, Roth CE. Hantavirus infection: a review and global update. J Infect Dev Ctries 2008; 2: 3–23. 19736383 10.3855/jidc.317

[bib36] Leroy EM, Kumulungui B, Pourrut X et al. Fruit bats as reservoirs of Ebola virus. Nature 2005; 438: 575–576. 16319873 10.1038/438575a

[bib37] Pourrut X, Kumulungui B, Wittmann T et al. The natural history of Ebola virus in Africa. Microb Infect 2005; 7: 1005–1014. 10.1016/j.micinf.2005.04.006 16002313

[bib38] Weingartl HM, Embury-Hyatt C, Nfon C et al. Transmission of Ebola virus from pigs to non-human primates. Sci Rep 2012; 2: 811. 23155478 10.1038/srep00811 PMC3498927

[bib39] Roth A, Mercier A, Lepers C et al. Concurrent outbreaks of dengue, chikungunya and Zika virus infections-an unprecedented epidemic wave of mosquito-borne viruses in the Pacific 2012-2014. Euro Surveill 2014; 19: 20929. 25345518 10.2807/1560-7917.es2014.19.41.20929

[bib40] Favoretto S, Araujo D, Oliveira D et al. First detection of Zika virus in neotropical primates in Brazil: a possible new reservoir. BioRxiv 2016, doi: http://www.biorxiv.org/content/early/2016/04/20/049395.

[bib41] Dick G. Zika virus (II). Pathogenicity and physical properties. Trans R Soc Trop Med Hyg 1952; 46: 521–534. 12995441 10.1016/0035-9203(52)90043-6

[bib42] Marano G, Pupella S, Vaglio S et al. Zika virus and the never-ending story of emerging pathogens and transfusion medicine. Blood Transfus 2016; 14: 95–100. 26674815 10.2450/2015.0066-15 PMC4786129

[bib43] Ogden NH, Bouchard C, Kurtenbach K et al. Active and passive surveillance and phylogenetic analysis of *Borrelia burgdorferi* elucidate the process of Lyme disease risk emergence in Canada. Environ Health Perspect 2010; 118: 909–914. 20421192 10.1289/ehp.0901766 PMC2920908

[bib44] Barbour AG, Maupin GO, Teltow GJ et al. Identification of an uncultivable *Borrelia* species in the hard tick *Amblyomma americanum*: possible agent of a Lyme disease-like illness. J Infect Dis 1996; 173: 403–409. 8568302 10.1093/infdis/173.2.403

[bib45] Moriarty TJ, Norman MU, Colarusso P et al. Real-time high resolution 3D imaging of the lyme disease spirochete adhering to and escaping from the vasculature of a living host. PLoS Pathog 2008; 4: e1000090. 18566656 10.1371/journal.ppat.1000090 PMC2408724

[bib46] Tilly K, Rosa PA, Stewart PE. Biology of infection with *Borrelia burgdorferi*. Infect Dis Clin North Am 2008; 22: 217–234. 18452798 10.1016/j.idc.2007.12.013 PMC2440571

[bib47] Almeida MA, Cardoso JDC, dos Santos E et al. Surveillance for yellow fever virus in non-human primates in Southern Brazil, 2001–2011: a tool for prioritizing human populations for vaccination. PLoS Negl Trop Dis 2014; 8: e2741. 24625681 10.1371/journal.pntd.0002741 PMC3953010

[bib48] Monath TP. Yellow fever: an update. Lancet Infect Dis 2001; 1: 11–20. 11871403 10.1016/S1473-3099(01)00016-0

[bib49] Roetzer A, Diel R, Kohl TA et al. Whole genome sequencing versus traditional genotyping for investigation of a *Mycobacterium tuberculosis* outbreak: a longitudinal molecular epidemiological study. PLoS Med 2013; 10: e1001387. 23424287 10.1371/journal.pmed.1001387 PMC3570532

[bib50] Sanger F, Nicklen S, Coulson AR. DNA sequencing with chain-terminating inhibitors. Proc Natl Acad Sci 1977; 74: 5463–5467. 271968 10.1073/pnas.74.12.5463 PMC431765

[bib51] Dapp MJ, Heineman RH, Mansky LM. Interrelationship between HIV-1 fitness and mutation rate. J Mol Biol 2013; 425: 41–53. 23084856 10.1016/j.jmb.2012.10.009 PMC3534800

[bib52] Taylor LH, Latham SM, Mark E. Risk factors for human disease emergence. Philos Trans R Soc Lond B Biol Sci 2001; 356: 983–989. 11516376 10.1098/rstb.2001.0888 PMC1088493

[bib53] Okinaka R, Cloud K, Hampton O et al. Sequence, assembly and analysis of pX01 and pX02. J Appl Microbiol 1999; 87: 261–262. 10475962 10.1046/j.1365-2672.1999.00883.x

[bib54] Miosge LA, Field MA, Sontani Y et al. Comparison of predicted and actual consequences of missense mutations. Proc Natl Acad Sci 2015; 112: 5189–5198. 26269570 10.1073/pnas.1511585112 PMC4577149

[bib55] Šmajs D, Zobaníková M, Strouhal M et al. Complete genome sequence of *Treponema paraluiscuniculi*, strain Cuniculi A: the loss of infectivity to humans is associated with genome decay. PLoS One 2011; 6: e20415. 21655244 10.1371/journal.pone.0020415 PMC3105029

[bib56] Morozova O, Marra MA. Applications of next-generation sequencing technologies in functional genomics. Genomics 2008; 92: 255–264. 18703132 10.1016/j.ygeno.2008.07.001

[bib57] Prakash T, Taylor TD. Functional assignment of metagenomic data: challenges and applications. Brief Bioinform 2012; 13: 711–727. 22772835 10.1093/bib/bbs033 PMC3504928

[bib58] Hofmann W, Schubert D, LaBonte J et al. Species-specific, postentry barriers to primate immunodeficiency virus infection. J Virol 1999; 73: 10020–10028. 10559316 10.1128/jvi.73.12.10020-10028.1999 PMC113053

[bib59] Hoye BJ, Munster VJ, Nishiura H et al. Reconstructing an annual cycle of interaction: natural infection and antibody dynamics to avian influenza along a migratory flyway. Oikos 2011; 120: 748–755.

[bib60] Roche B, Rohani P, Dobson AP et al. The impact of community organization on vector-borne pathogens. Am Nat 2012; 181: 1–11. 23234841 10.1086/668591

[bib61] Zeppelini CG, de Almeida AMP, Cordeiro-Estrela P. Zoonoses as ecological entities: a case review of plague. PLoS Negl Trop Dis 2016; 10: e0004949. 27711205 10.1371/journal.pntd.0004949 PMC5053604

[bib62] Kao RR, Haydon DT, Lycett SJ et al. Supersize me: how whole-genome sequencing and big data are transforming epidemiology. Trends Microbiol 2014; 22: 282–291. 24661923 10.1016/j.tim.2014.02.011 PMC7125769

[bib63] Tang JW. The effect of environmental parameters on the survival of airborne infectious agents. J R Soc Interface 2009; 6: 737–746. 10.1098/rsif.2009.0227.focus PMC2843949 19773291

[bib64] Dietze R, Barros GB, Teixeira L et al. Effect of eliminating seropositive canines on the transmission of visceral leishmaniasis in Brazil. Clin Infect Dis 1997; 25: 1240–1242. 9402389 10.1086/516096

[bib65] Ezenwa VO, Jolles AE. Opposite effects of anthelmintic treatment on microbial infection at individual versus population scales. Science 2015; 347: 175–177. 25574023 10.1126/science.1261714

[bib66] Menge BA, Sutherland JP. Species diversity gradients: synthesis of the roles of predation, competition, and temporal heterogeneity. Am Nat 1976; 110: 351–369.

[bib67] McCafferty J, Mühlbauer M, Gharaibeh RZ et al. Stochastic changes over time and not founder effects drive cage effects in microbial community assembly in a mouse model. ISME J 2013; 7: 2116–2125. 23823492 10.1038/ismej.2013.106 PMC3806260

[bib68] Lindenfors P, Nunn CL, Jones KE et al. Parasite species richness in carnivores: effects of host body mass, latitude, geographical range and population density. Glob Ecol Biogeogr 2007; 16: 496–509.

[bib69] Giardina AR, Schmidt KA, Schauber EM et al. Modeling the role of songbirds and rodents in the ecology of Lyme disease. Can J Zool 2000; 78: 2184–2197.

